# The Ability of Saudi Parents’ To Detect Early Language Delay in Their Children: A Study in Primary Health Care Centers, King Abdulaziz Medical City, Riyadh, Saudi Arabia

**DOI:** 10.7759/cureus.21448

**Published:** 2022-01-20

**Authors:** Maha H Alakeely, Howaida Alabbasi, Lama Alohali, Aida Aldughaither

**Affiliations:** 1 Family Medicine, King Abdulaziz Medical City, Ministry of National Guard Health Affairs, Riyadh, SAU; 2 Research Center, King Abdullah International Medical Research Centre, Riyadh, SAU; 3 Speech Pathology, Child and Adolescent Psychiatry and Behavioral Science, King Abdullah Specialist Children's Hospital, Ministry of National Guard Health Affairs, Riyadh, SAU; 4 Family Medicine, College of Medicine, King Saud Bin Abdulaziz University for Health Sciences, Riyadh, SAU

**Keywords:** parents, early detection, well-baby, delay, language

## Abstract

Purpose

Our study aims to assess parents’ ability to detect early language delay in their children in association with related demographic and environmental factors to help in predicting its risk.

Methods

A cross-sectional study was conducted at three main primary health care centers at National Guard Health Affairs, Riyadh, Saudi Arabia. Participating parents were asked if they think their children have language delay and if they were able to detect it early. Then, validated age-appropriate screening tools were administered to assess the child’s language development. The parents' answers regarding their child’s language development were then compared to the screening tool assessment results.

Participants

A total of 250 parents attending a well-baby clinic for their children’s routine vaccinations participated in the study after informed consent was obtained.

Results

Language delay was more prominent in the one-year-old age group (26.7%). In addition, children who were not breastfed were significantly more likely to have language delays (P-value 0.014). The parents’ ability to detect language delay varied among the different age groups. Fifty-seven point one percent (57.1%) of children aged two years old and 61.5% of children aged five years old who were found to have language delay were not noticed by their parents (P-value 0.03, 0.02).

Conclusion

Parents showed a lack of ability to detect language delay early. Increasing their awareness of the typical language development milestones and the importance of early intervention is very important to minimize the consequences of late intervention.

## Introduction

Language progression and maturation is an essential part of typical development [1]. Language is defined as “the conceptual processing of communication,” which includes receptive and expressive language [1-2]. Language development progresses through cooing, bubbling, and then from simple words to phrases and more complex sentences [3]. Although the rate of this progress may vary from one child to another, standardized screening milestones are used to recognize any delays and determine the need for further assessment [4]. Language delay refers to any late development of age-appropriate language skills in the absence of any other justifiable causes like hearing impairment, emotional problems, or cognitive deficits [5].

Several studies from different countries demonstrated the prevalence of language delay among children. In the US, for example, language delay is estimated to be found in 8-9% of young children [6]. In the UK, six studies estimated the prevalence in children aged two to five years old to be between 5% and 12% [7]. In India, a study published in 2019 found language delay in about 2.53% of children aged one to 12 years old [8]. In Iraq, the frequency of language delay was 11.9% among children aged less than seven years old [9]. Locally, in the Kingdom of Saudi Arabia and among preschool children, the overall prevalence of language delay was dramatically higher, reaching up to 24.5% [10].

Many factors could contribute to a higher risk of language delay among children. According to a study in Saudi Arabia, groups with a higher risk included males, only children, children with a family history of speech delay, children whose mothers had only high school education, children birthed with a forceps delivery, children with inadequate time spent with their mother and other children, and children that spend more time alone or in front of a screen [10]. In addition, there are some medical risk factors such as having a history of birth asphyxia, seizure disorder, and oropharyngeal deformity [8].

Although a good number of children with an early language delay will catch up as they age, some will have persistent difficulties with their language skills [11]. Early detection and intervention can prevent poor outcomes as a result of language delay and improve the quality of life for the child [12-13]. Some parents are not aware of the typical language milestones. In contrast, some parents recognize the missed milestone but assume that the child will “grow out of it” and catch up with time [11]. This assumption leads to parents seeking medical advice later, which means the precious time of early intervention is lost. Our study aims to assess parents’ ability to detect language delays early, measure the percentage of language delays among children attending well-baby clinics, and describe the demographical and environmental factors of parents and their children that could increase the risk for language delay in different age groups.

## Materials and methods

Study design, area, and setting

This cross-sectional study was conducted at the well-baby clinics of three of the Ministry of National Guard Health Affairs (MNGHA) primary health care centers. MNGHA has many primary health care centers distributed around different regions of Saudi Arabia. The Health Care Specialty Centre (HCSC), National Guard Comprehensive Specialized Centre (NGCSC), and King Abdulaziz City Housing (Iskan Yarmouk) center are the main primary health care centers in Riyadh, covering a large population with easy accessibility. Iskan Yarmouk clinics and HCSC serve patients living in the eastern region of Riyadh while NGCSC serves patients living in the northern region. All centers provide general outpatient care with different specialties. Well-baby clinics are responsible for children's vaccines and general well-being. The age of children visiting the clinic is based on the Saudi vaccine schedule starting at two months with regular visits until the age of six. If any problem is noticed during these visits, then the child will be referred for further investigation and management.

Identification of study participants

The questionnaire was completed by parents attending the well-baby clinic for their children’s routine vaccinations. Children who were Saudi nationals, speaking the Arabic language, aged one, one and a half, two, three, and five years were included in the study. Exclusion criteria were applied if the child had oropharyngeal deformities like cleft lip palate or hearing problems or was known to have autism.

Data collection

A convenience sampling technique was used. Two-hundred fifty parents, either the child's mother or father, who were attending the well-baby clinic for their children’s vaccinations were invited to participate in the study. Data were collected by assigned nurses who are working in the well-baby clinics. After the study was explained, parents were asked to sign an informed consent form if they agreed to participate in the study. Data were collected through a self-administered questionnaire distributed to 250 parents. The questionnaire contained three sections. The first included questions related to the child’s age, gender, number of siblings, birth order, duration of breastfeeding, and other possible factors associated with language delay. The second section included questions about the parents’ age, educational levels, income, and occupation status. The remaining questions in this section focused on whether the parents noticed any language delay in their child and understood the possible risk factors associated with language delay. The last section included screening questions according to the child’s age. The Centers for Disease Control and Prevention (CDC)-validated Arabic language milestone screening test was administered to parents based on the child’s age. The validity of the questionnaire was assessed by two family physician consultants, two pediatricians, and two speech therapists. After applying their revisions, the completed questionnaire was checked and pretested for clarity and suitability in a small pilot study of ten parents. When administered to the full test group, if the parents answered in the negative to more than 20% of the screening questions, the subject was categorized as abnormal and in need of further assessment by a speech and language pathologist. The parents' opinion of their child’s language development was then compared to the results of the CDC assessment.

Ethical considerations

Ethical approval was granted by the ethical review board of King Abdullah International Medical Research Center, Riyadh, KSA, Reference #: IRBC/0253/20. Participant consent was obtained through a consent form. The collected data were stored in a secure place where only the principal investigator and co-investigators had access. The data did not contain any identifying information. The names of the participants were coded and stored in a computer with password protection.

Data analysis

The data were presented as the mean±standard deviation for continuous variables (e.g., age of parents) and frequencies (percentages) for categorical variables (e.g., age of children, gender, education level, occupation, etc.) A chi-square test or Fisher’s exact test was used to compare categorical variables (e.g., gender, birth order, family income, education level, and language delay). A p-value of <0.05 was considered statistically significant. Data were entered and analyzed using SPSS software version 23 (IBM Corp., Armonk, NY).

## Results

Parents' demographics

Table 1 shows the demographic characteristics of parents. The questionnaire was completed by a total of 250 parents, either the child's mother or father. The participated parent filled also his/her partner's related demographic questions. Two-hundred seventeen (86.6%) of the parents who completed the questionnaire were the mothers. The mean age of the mothers was 31±4.41 years. Two-hundred one (80.4%) of the mothers were housewives and 122 (48.8%) had completed college-level education and above. The mean age of the fathers was 36±7.23 years. One-hundred seventy-two fathers (68.8%) had completed high school education, 241 (96.4%) were employed, and 140 (56.0%) had a monthly family income between 5,000 and 10,000 SR.

**Table 1:** Demographic characteristics of the parents (n=250)

Characteristics		N (%)
Relation to the child	Mother	217 (86.8)
	Father	33 (13.2)
Age of the mother, y	Below 25	22 (8.8)
	Between 25–35	169 (67.6)
	More than 35	59 (23.6)
Age of the father, y	Below 25	3 (1.2)
	Between 25–35	130 (52.0)
	More than 35	59 (46.8)
Educational level of the mothers	Illiterate (no education)	6 (2.4)
	School education ( elementary, intermediate, or high school)	122 (48.8)
	University degree or above	122 (48.8)
Education level of the fathers	School education ( elementary, intermediate, or high school)	172 (68.8)
	University degree or above	78 (31.2)
Occupation of the mother	Employed	49 (19.6)
	Housewife	201 (80.4)
Occupation of the father	Employed	241 (96.4)
	Unemployed	9 (3.6)
Family monthly income, Saudi Riyal	Less than 5000	38 (15.2)
	5000–10000	140 (56)
	More than 10000	72 (28.8)

Children’s demographics

Table 2 shows the demographic characteristics of the children. A total of 250 children aged one, one and a half, two, three, and five were included in the study. One-hundred twenty-four (56.8%) were males. One-hundred sixty-seven (66.80%) were breastfed, with 160 (64%) breastfed for a duration of less than six months. Two-hundred forty-seven (98.80%) lived with both parents, and 192 (76.80%) lived with extended family. A nanny or someone other than the parents took care of 49 (19.6%) participants. One-hundred eighty-eight (75.20%) had siblings, with 84 (33.6%) being the first child. Two-hundred thirty-four (93.60%) of the children were not attending nursery or school. Eighty-two (33.06%) of the parents reported screen time of their children of less than one hour per day, mostly with smartphones (56.7%).

**Table 2:** Demographic characteristics of the children (n=250)

Characteristics		N (%)
Age, y	1	71 (28.4)
	1.5	40 (16.0)
	2	63 (25.2)
	3	32 (12.8)
	5	44 (17.6)
Gender	Female	108 (43.2)
	Male	142 (56.8)
Living with both parents	Yes	247 (98.8)
	No	3 (1.2)
Living with extended family	Yes	58 (23.2)
	No	192 (76.8)
Nanny or someone else is taking care of the child	Yes	49 (19.6)
	No	201 (80.4)
Siblings	Yes	188 (75.2)
	No	62 (24.8)
Birth order	First child	84 (33.7)
	Second child	48 (53.0)
	Third child	50 (20.1)
	Fourth or more	67 (26.8)
Attending nursery	Yes	16 (6.4)
	No	234 (93.6)
Breastfeeding	Yes	167 (66.8)
	No	83 (33.2)
Breastfeeding duration	Less than 6 months	160 (64.0)
	6 to less than 12 months	40 (16.0)
	12 to 24 months	50 (20.0)
Screen time	No screen time	47 (19.0)
	Not more than 1 hour	82 (33.1)
	Within 2 hours	52 (21.0)
	More than 2 hours	67 (27.0)
Devices the child spend time using	Tablets	90 (36.0)
	Smartphone	144 (57.6)
	TV	97 (38.8)
	Computer	10 (4.0)

The percentage of language delay among participating children

Among participants who were screened, language delay was detected in 26.7% of children aged one-year-old, 26.3% of children aged one and a half years old, 22.2% of children aged two years old, 22.6% of children aged three years old, and 31.0% of children aged five years old (Table 3).

**Table 3:** Language delay among participants (n=250)

Age of the child, y (N)	Normal language development N (%)	Language delay N (%)
1 (75)	55 (73.3)	20 (26.7)
1.5 (38)	28 (73.7)	10 (26.3)
2 (63)	49 (77.8)	14 (22.2)
3 (31)	24 (77.4)	7 (22.6)
5 (42)	29 (69.0)	13 (31.0)

Parent’s ability to detect language delay

To assess the parent’s ability to detect their children’s language delay, the answers to the question “Do you think your child has a language delay?” were compared to the screening tool results. The results were varied among the different age groups (Table 4). Significantly, children aged two and five years old, who were determined to have a language delay by the assessment tool were not picked up by their parents (P-value 0.03, 0.02).

**Table 4:** Parent’s ability to detect language delay

Age, y	Language assessment	Do you think your child has a language delay?		P-value
		Yes	No	
1	No language delay	7 (12.7%)	48 (87.3%)	0.55
	Language delay	2 (10.0%)	18 (90.0%)	
1.5	No language delay	1 (3.6%)	27 (96.4%)	0.16
	Language delay	2 (20%)	8 (80.0%)	
2	No language delay	3 (6.1%)	46 (93.9%)	0.003
	Language delay	6 (42.9%)	8 (57.1%)	
3	No language delay	0 (0.00%)	24 (100%)	0.008
	Language delay	3 (42.9%)	4 (57.1)	
5	No language delay	0 (0.00%)	29 (100%)	0.002
	Language delay	5 (38.5%)	8 (61.5%)	

Demographical and environmental factors of children that could predict higher risk for language delay in the one to five-year age groups

Table 5 presents the associations between the demographic characteristics of the participants and language delay. Among the variables we assessed, more language delay was noticed among children who were not breastfed (P-value 0.014). There was no significant difference in the presence of language delay and birth order, screen time, a specific gender, children not living with extended family, not attending nursery, and no siblings (P-value = 0.06, 0.500, 0.202, 0.871, 0.212, 0.768, 0.188, respectively).

**Table 5:** Association between children's demographic characteristics and language delay

Demographic characteristics		No language delay N (%)	Language delay N (%)	P-value
Gender	Female	77 (71.3)	31 (28.7)	0.202
	Male	109 (76.8)	33 (23.2)	
Age, y	1	53 (74.6)	18 (25.4)	0.871
	1.5	30 (75)	10 (25)	
	2	48 (76.2)	15 (23.8)	
	3	25 (78.1)	7 (21.9)	
	5	30 (68.2)	14 (31.8)	
Living with both parents	Yes	184 (74.5)	63 (25.5)	0.590
	No	2 (66.7)	1 (33.3)	
Living with extended family	Yes	46 (79.3)	12 (20.7)	0.212
	No	140 (72.9)	52 (27.1)	
Nanny	Yes	37 (75.5)	12 (24.5)	0.501
	No	149 (74.1)	52 (25.9)	
Siblings	Yes	143 (76.1)	45 (23.9)	0.188
	No	43 (69.4)	19 (30.6)	
Birth order	First child	62 (73.8)	22 (26.2)	0.065
	Second	29 (60.4)	19 (39.6)	
	Third	41 (82.0)	9 (18.0)	
	Fourth or more	53 (79.1)	14 (20.9	
Attending nursery	Yes	13 (81.2)	3 (18.8	0.768
	No	173 (73.9)	61 (26.1)	
Breastfeeding	Yes	132 (79.0)	35 (21.0)	0.014
	No	54 (65.1)	29 (34.9)	
Duration of Breastfeeding	Less than 6 months	115 (71.9)	45 (28.1)	0.458
	6 to less than 12 months	31 (77.5)	9 (22.5)	
	12 to 24 months	40 (80.0)	10 (20.0)	
Screen time	No screen time	33 (70.2)	14 (29.8)	0.500
	Not more than 1 hour	60 (73.2)	22 (26.8)	
	Within 2 hours	43 (82.7)	9 (17.3)	
	More than 2 hours	50 (74.6)	17 (25.4)	

Demographical and environmental factors of parents who could predict higher risk for language delay in children in the one to five-year age groups

Among the variables of age, level of education, occupation of parents, and family income, none were significantly associated with an increase in language delay among children participating in the study.

## Discussion

Our study aimed to assess parents’ ability to detect language delay early, as it is a prevalent issue in Saudi Arabia [10]. A 2020 study found that a delay in communication skills was one of the most prevalent developmental delays in Saudi Arabian preschool children [14]. The very first few years of a child’s life are very important for language development. Parents play a major role in stimulating this skill [15]. A study done in 2019 found that inadequate stimulation by parents is one of the main familial causes of language delay [8]. Using gestures while talking to the child, frequent naming of things around the child’s environment, and exposing the child to a variety of experiences are all examples of how parents can stimulate receptive language. Expressive language, on the other hand, can be stimulated by encouraging the child to imitate sounds, using greetings, naming things in the environment, and saying whatever they want with the full attention and response of their parents [16].

As for parents’ ability to detect a language delay in their children, our results demonstrated higher rates of undetected language delay in children aged one to two years old. This result might be due to the complexity of language assessment in earlier ages, as language assessment encompass word production and non-verbal communication skills such as eye gaze [17]. Also, parents might think their children will outgrow it or feel that a communication problem is not a high priority [18]. However, it is noteworthy to point out the significance of early detection of language delay, as studies show that speech and language delays that go untreated can continue in 40%-60% of the children and can affect them in both their childhood and adulthood [19-20]. As children, they are at an increased risk of having poor school performance, reading skills, socialization, and attention difficulties [1-2]. As adults, a higher rate of unemployment and low income were reported compared to people with no language delay [7].

Among the variables that have been assessed and could predict a higher risk for language delay, language delay was noticed among children who were not breastfed. Data on this topic are mixed, as some studies suggest that children who are not breastfed are more prone to language delay [21-22]. Some studies revealed no association between breastfeeding and language delay [23]. Birth order was not found to be associated with language delay but some studies suggest that first-born children might have better language skills than later-born siblings [24-25].

In our study, no significant association was found between language delay and a specific gender. However, research suggests that males are at a greater risk for delayed language development than females [26]. No significant difference was also found among children not attending nursery school or having no siblings and language delay. Despite that, children who receive minimal language stimulation tend to be delayed in language development [27]. Finally, our data found no association between language delay and screen time. However, some studies suggest that excessive use of screen time may lead to language delays in children [28-30].

Study strength and limitations

To the best of our knowledge, this is the first study conducted in Saudi Arabia to assess parents’ ability to detect language delays in their children. The result of our study may help raise awareness and shed light on the importance of educating parents about normal developmental milestones and when to seek medical advice. The study involved a self-administered questionnaire in which report bias was possible. Our study was conducted in only three primary health care centers under one tertiary hospital in Riyadh. Therefore, this study does not represent the entire population of Saudi Arabia. Due to the COVID-19 outbreak, we experienced difficulties with data collection. There was a decrease in the number of parents bringing their children to well-baby clinics. Furthermore, some parents refused to participate, as they were afraid to stay in the clinic longer or touch papers or pens.

## Conclusions

The result of this study demonstrated a lack of parents’ ability to detect language delays in their children, which may lead to a delay in seeking medical advice and early intervention. Parents’ awareness and education regarding the early symptoms of language delay and when to seek medical help is crucial for early detection, intervention, and better quality of life for the children’s future. In addition, further research is needed to shed light on this issue and explore more factors related to late diagnosis and management of language delay.
